# Cannabis based medicines and cannabis dependence: A critical review of issues and evidence

**DOI:** 10.1177/0269881120986393

**Published:** 2021-02-17

**Authors:** Anne K Schlag, Chandni Hindocha, Rayyan Zafar, David J Nutt, H Valerie Curran

**Affiliations:** 1 Drug Science, St Peter’s House, London, UK; 2 Neuropsychopharmacology Unit, Centre for Psychiatry, Imperial College London, London, UK; 3 Clinical Psychopharmacology Unit, University College London, London, UK; 4 Translational Psychiatry Research Group, Research Department of Mental Health Neuroscience, University College London, London, UK; 5 University College Hospital National Institute of Health Research (NIHR) Biomedical Research Centre, London, UK

**Keywords:** Medical cannabis, tetrahydrocannabinol, cannabidiol, dependence, cannabis use disorder

## Abstract

Cannabis has been legalised for medical use in an ever-increasing number of countries. A growing body of scientific evidence supports the use of medical cannabis for a range of therapeutic indications. In parallel with these developments, concerns have been expressed by many prescribers that increased use will lead to patients developing cannabis use disorder. Cannabis use disorder has been widely studied in recreational users, and these findings have often been projected onto patients using medical cannabis. However, studies exploring medical cannabis dependence are scarce and the appropriate methodology to measure this construct is uncertain. This article provides a narrative review of the current research to discern if, how and to what extent, concerns about problems of dependence in recreational cannabis users apply to prescribed medical users. We focus on the main issues related to medical cannabis and dependence, including the importance of dose, potency, cannabinoid content, pharmacokinetics and route of administration, frequency of use, as well as set and setting. Medical and recreational cannabis use differs in significant ways, highlighting the challenges of extrapolating findings from the recreational cannabis literature. There are many questions about the potential for medical cannabis use to lead to dependence. It is therefore imperative to address these questions in order to be able to minimise harms of medical cannabis use. We draw out seven recommendations for increasing the safety of medical cannabis prescribing. We hope that the present review contributes to answering some of the key questions surrounding medical cannabis dependence.

## Introduction

### What lessons can we learn from history?

Cannabis is arguably the world’s oldest medicine. It has been used medically for millennia especially in China and India. It was introduced into Western medicine in the mid-19th century as various tinctures and extracts. The medical use of cannabis in the West declined in the early 20th century, partly due to extreme variations in effects depending on where, how, when and what strain of plant material had been grown. We now understand these variations as deriving from different combinations and concentrations of over 140 cannabinoids and more than 100 different terpenoids found in different strains of the cannabis plant.

A new cycle has begun for the use of cannabis derivatives as medicines, this time with more consistency than in the past. Interest in the therapeutic benefits of medical cannabis has grown rapidly in the past 20 years, with patients using cannabinoids to treat a variety of conditions, from chronic pain, cancer and neurological disorders to anxiety and sleep disorders (Schlag et al., 2020; United Patients’ Alliance [UPA], 2018). The structures of chemical compounds derived from cannabis are now known, their mechanisms of action in the nervous system are being elucidated as we learn more about our endogenous cannabinoid system, and treatment effectiveness and safety are increasingly being evaluated scientifically.

Clearly, a more widespread use of cannabis is for recreation and pleasure. In reality, the divide between recreational use and medical use of a drug is quite blurred, and history is dotted with drugs that have been used for both purposes. Opiates have been used medically to reduce pain and recreationally for pleasure; benzodiazepines to reduce anxiety and promote sleep for patients and recreational users alike. Alcohol, our most common recreational drug, was once used medically as an anaesthetic and analgesic before more effective drugs emerged. Amphetamines have been used medically for Attention Deficit Hyperactivity Disorder (ADHD) (and appetite suppression) and recreationally as ‘speed’; ketamine has been used since 1964 as anaesthetic, since the 1990s as a recreational drug and since 2019 as a licensed, rapid-acting anti-depressant for individuals resistant to other treatments.

We can learn some lessons from how medical use of opiates, benzodiazepines and other psychoactive drugs lead to dependence in some patients but not others. However, medical cannabis use may well differ, in part because cannabis is a potential treasure chest of many pharmacologically different medicines. It might also differ in that some cannabinoids like tetrahydrocannabinol (THC) in higher doses and frequent usage promote dependence whilst cannabidiol (CBD), may have anti-addictive properties (Freeman et al., 2020; Morgan et al., 2010). This means that carefully balancing the cannabinoid content of cannabis-based medicines could potentially block the development of dependence.

### Aims and rationale of the current review

Research on medical cannabis dependence is still in its infancy. Most of the studies on cannabis dependence have been conducted in relation to recreational use, which substantially differs from the medical application of cannabis. The differences between medical and recreational cannabis use and users highlight the challenges of simply extrapolating findings from the recreational cannabis literature. Many questions about the potential for medical cannabis use to lead to dependence remain to be answered. It is imperative to address these questions in order to be able to minimise harms of medical cannabis use. Here, we offer a narrative review of the literature and consider to what extent research on recreational cannabis dependence might be applied to medical cannabis dependence.

### What are cannabis-based medicines?

In recent decades, many US states and over 20 countries globally, have instigated regulatory regimes of medical use of the plant and the World Health Organization (WHO) has now accepted its medical use (World Health Organization [WHO], 2019). However, there are significant differences in the way that medical cannabis policies are enacted and there are no international standards on the best way to regulate this. Whilst appreciating that definitions, regulations and subsequent access to medical cannabis differ between countries (Schlag, 2020), the current article adopts a UK perspective for these specifics only, although the overall conclusions and recommendations will have wider applicability.

There is often uncertainty about what exactly defines cannabis, a cannabinoid, or THC, as well as the different formulations available. It is vital to distinguish between CBD (not a controlled drug in the UK) and formulations of CBD plus THC to ensure a clear understanding of the distinctions in active ingredients and in formulations as these relate to specific applications. Freeman et al. (2019a) provide a helpful summary, highlighting that cannabis is not one medicine but rather a whole family of medicines. We focus on cannabis-based medicinal products (CBMPs) as defined by the Misuse of Drugs (Amendments) (Cannabis and Licence Fees) (England, Wales and Scotland) Regulations 2018:

[A] cannabis-based product for medicinal use in humans means a preparation or other product. . . which a) is or contains cannabis, cannabis resin, cannabinol or a cannabinol derivative (not being dronabinol or its stereoisomers); (b) is produced for medicinal use in humans; and (c) is (i) a medicinal product, or (ii) a substance or preparation for use as an ingredient of, or in the production of an ingredient of, a medicinal product.

(National Institute for Health and Care Excellence [NICE], 2019)

#### Cannabis-based medicines in the UK

Currently there are four licenced CBMPs that can be prescribed by specialist doctors. These are:

1. Two THC-based medicines: dronabinol – licensed for appetite loss in acquired immune deficiency syndrome (AIDS) and as an anti-emetic in chemotherapy and nabilone licensed for nausea in individuals receiving chemotherapy.
2. Combined THC: CBD medicines: Sativex – for muscle spasticity in multiple sclerosis
3. CBD-based medicines: Epidiolex (99.8% CBD with less than 0.1% THC) – for two rare childhood epilepsies (Lennox-Gastaut and Dravets syndrome) (NICE, 2019).

There are a multitude of other unlicensed cannabis-based products (e.g. oils, herbal cannabis) that are produced to Good Manufacturing Practices (GMP) standard and so can now be prescribed in the UK.

### Addiction vs dependence: chewing the ’CUD’

Addiction, dependence, abuse, substance use disorder – there is a terminological quagmire in struggling to de-stigmatise by changing names across time. Here, we define addiction as an acquired, chronic, relapsing disorder that is characterised by a powerful motivation to continually engage in an activity despite persistent negative consequences. Dependence on a drug can occur without these drug-seeking activities or such persistent negative consequences. Dependence is therefore a more appropriate term when referring to medicinal cannabis use.

Addictive drugs can all cause similar changes by hijacking brain circuits underpinning reward, salience, impulsivity, compulsivity, learning and memory (Everitt et al., 2016; Goldstein and Volkow, 2011; Koob et al., 2010; Nestler, 2005). The result is a dysregulation between regulatory executive ‘top-down’ control and ‘bottom-up’ reward and emotional drives. This imbalance gradually leads to a loss of control over impulsive and then compulsive drug seeking behaviours (Droutman et al., 2015).

Clinical problems associated with cannabis use were once diagnosed as either ’cannabis abuse’ or ’cannabis dependence’ in the *Diagnostic and Statistical Manual for Mental Disorders*, fourth edition, text revision (DSM‑IV‑TR). In the most recent version (DSM‑5), these categories were amalgamated into a single diagnosis of ‘cannabis use disorder’ (CUD), as described in Table 1. DSM-5 defines CUD as: ’A problematic pattern of cannabis use leading to clinically significant impairment or distress, as manifested by at least two of the symptoms from the three lists in Table 1, occurring within a 12-month period’ (American Psychiatric Association [APA], 2013). Mild CUD is associated with having two or three of these symptoms, moderate CUD with four or five symptoms, and severe CUD with six or more symptoms. One advantage of combining abuse and dependence criteria is the provision of a clearer continuum between mild and severe presentations as previously all cases of dependence also met criteria for abuse. Another advantage is that dependence on a medicine is often a common and ‘normal’ situation. For example, stopping the use of a prescribed pain killer does not necessarily lead to drug-seeking behaviours but individuals may be dependent upon them for pain relief (Supplementary Table 1).

**Table 1.** Cannabis use disorder symptoms from *Diagnostic and Statistical Manual for Mental Disorders*, fourth edition, text revision (DSM‑IV‑TR) to DSM-5 with likely frequency of symptom based on the literature related to medical cannabis use and justification for likelihood.

The following symptoms are taken from the DSM-IV criteria for cannabis dependence	Likely frequency of symptom based on current literature review (not seen, rarely, common, unknown)	Evidence
Cannabis is often taken in larger amounts or over a longer period than was intended	Rarely	Dose will likely increase over time. ‘Start low-go slow’ and achieving optimal dose may be more than initially intended. Clinical trials of CBMPs have not evidenced this symptom, patients are often titrated up to a tolerable dose based on the individual’s physiological response to CBMPs (Robson, 2011).
		Amongst 801 patients seeking medical cannabis certification in Michigan, the daily (or almost daily use) users over the past three months, 97.2% (*n*=564) reported no dose-escalation over the past 6-month period. The same pattern of results was observed amongst weekly cannabis users. Reductions in use were more common that increases (Perron et al., 2019).
		During long-term follow-up of a clinical trial of dronabinol for neuropathic pain, 19 patients increased and 24 decreased their dose temporarily, 63 patients did not change their dose (Schimrigk et al., 2017).
There is a persistent desire or unsuccessful efforts to cut down or control cannabis use	Not seen	Canadian registry data show ½ patients stop after 6 months which argues against significant dependence and most who stop do not report problems (Government of Canada, 2019).
		If there was an occasion wherein it was deemed necessary to wean someone off medical cannabis and they presented with classic withdrawal symptoms i.e. insomnia, anxiety, irritability (unrelated to the condition trying to be treated), then this may become an issue
A great deal of time is spent in activities necessary to obtain cannabis, use cannabis, or recover from its effects	Not known	Taken as prescribed. Further investigation is required.
Important social, occupational, or recreational activities are given up or reduced because of cannabis use	Not seen	Social, recreational and occupational engagement may be improved if symptom improvement is great enough. Limited (anecdotal) research available.
Cannabis use is continued despite knowledge of having a persistent or recurrent physical or psychological problem that is likely to have been caused or exacerbated by cannabis	Not seen	Risks vs benefits need to be considered for medicines. Some physical/psychological side effects from medical cannabis may have to be tolerated in order to achieve main therapeutic aim e.g. reduction in seizures vs acute side effects when increasing dose. Trade-off is the same with most neuroleptic medications.
Tolerance, as defined by either (a) a need for markedly increased cannabis to achieve intoxication or desired effect or (b) a markedly diminished effect with continued use of the same amount of the substance	Rarely	Little is known about medical cannabis tolerance effects. No tolerance was observed with sativex (Wade et al., 2006) or dronabinol (Schimrigk et al., 2017). Tolerance can be avoided by prescribing low-dose THC, with CBD (MacCallum and Russo, 2018).
Withdrawal, as manifested by either (a) the characteristic withdrawal syndrome for cannabis or (b) cannabis is taken to relieve or avoid withdrawal symptoms Cannabis abuse symptoms from DSM-IV	Common	10/244 treated with dronabinol experienced transient withdrawal symptoms after cessation (Schimrigk et al., 2017). No withdrawal syndrome associated with CBD (Taylor et al., 2020).
		In a study of *n*=801 medical cannabis certification seeking chronic pain patients in Michigan, 67.8% reported at least one moderate or severe withdrawal symptom. Sleep difficulties was most common (50.3%), followed by anxiety (27.8%), irritability (26.7%), and appetite disturbance (25.2%) (Perron et al., 2019).
The following symptoms are taken from DSM-IV criteria for cannabis abuse		
Recurrent cannabis use resulting in a failure to fulfil major role obligations at work, school, or home	Unknown	Little research has been conducted on the ability to fulfil major role obligations after medical cannabis use. Anecdotal patient reports suggest it is quite likely that medical cannabis will have the opposite effect.
Continued cannabis use despite having persistent or recurrent social or interpersonal problems caused or exacerbated by the effects of cannabis	Unknown	Little research has been conducted on the social and interpersonal problems caused or exasperated by medical cannabis. Its quite likely that medical cannabis will have the opposite effect.
Recurrent cannabis use in situations in which it is physically hazardous	Unknown	Prescription leaflets advise patients to not use cannabis when it can be physically hazardous.
New symptom in DSM-5		
Craving, or a strong desire or urge to use cannabis	Unknown	Further research is required. Perron et al. (2019) craving was the 6th (/15) most endorsed withdrawal symptom.
		Whether craving occurs for the drug itself or symptom relief needs to be established.

DSM-IV: Diagnostic and Statistical Manual of Mental Disorders, Fourth Edition; CBD: cannabidiol; CBMP: cannabis-based medicinal product; THC: tetrahydrocannabinol.

For recreational users, the estimated chances of becoming dependent on cannabis after any lifetime exposure is 8.9%, which is considerably lower than for cocaine (20.9%), alcohol (22.7%) or tobacco (67.5%) (Lopez-Quintero et al., 2011). Nevertheless, the clinical need for treatment of cannabis dependence is substantial and increasing across North America, Europe and Oceania (United Nations Office on Drugs and Crime [UNODC], 2015). In Europe, cannabis now accounts for more first‑time entrants to drug treatment services than any other illicit drug (EMCDDA, 2015). Globally, the United Nations reports that cannabis dependence is now the primary reason for drug treatment across the world (39%), surpassing numbers receiving opioid treatment (33%) (UNODC, 2017).

A specific cannabis withdrawal syndrome – one aspect of dependence – is well-recognised and affects around 50% of daily users and typically begins 1–2 days after cessation, peaks at 2–6 days and remits at 1–2 weeks (Budney et al., 2004). Prominent symptoms include craving, sleep problems, nightmares, anger, irritability, dysphoria and nausea (Allsop et al., 2011). This can be precipitated by the Cannabinoid 1 receptor (CB1) receptor antagonist rimonabant (Steward and McMahon, 2010). Cannabis withdrawal symptoms in humans correlate with reductions in brain CB1 receptor availability during acute abstinence (D’Souza et al., 2016).

#### Cannabis, THC and dependence

The main cannabinoid with psychoactive properties is THC, which was made a Schedule 1 drug under the United Nations *Convention on Psychotropic Substances* as it has the capacity to produce a state of dependence (United Nations, 1971). A wealth of pharmacological evidence supports the role of THC as the addictive agent within cannabis. THC produces the effects that recreational cannabis users seek; they report liking it and wanting more and this increases with acute THC dosage (Curran et al., 2002). In addition, cannabis with higher THC content (for example, 5% versus 2%) produces stronger reinforcement in human choice paradigms (Justinova et al., 2005). THC binds to the CB1 receptor to exert its psychoactive effect and CB1 receptor antagonists have been found to precipitate withdrawal in mice that have been exposed to THC (Cook et al., 1998; Steward and McMahon, 2010). In addition, studies have found that acute administration of THC is reinforcing in humans whether or not CBD is co-administered (Haney et al., 2016; Hindocha et al., 2015). As the reinforcement of drug use is considered to be one component in the transition from voluntary to compulsive use (Everitt and Robbins, 2016), these findings suggest that cannabis with high THC content increases vulnerability to dependence. Indeed, clinically, increases in the number of new entrants into treatment for cannabis use disorder have followed increases in THC potency (Freeman et al., 2018). The risk of recreational cannabis dependence is more common with high potency THC strains with a low CBD content, large amounts consumed, high frequency use (heavy, daily) and with starting use early in adolescence (Curran et al., 2016).

#### The role of CBD

CBD has no abuse potential (WHO, 2018) which is why it has never been a controlled drug in the UK. CBD has a complex range of pharmacological actions. For example, although CBD has a low affinity for the CB1 receptor, it can attenuate CB1 receptor agonist effects in the brain even at low concentrations (Straiker et al., 2018; Tham et al., 2018). CBD also reduces the cellular re-uptake and hydrolysis of the brain’s endogenous cannabinoid anandamide (AEA; also known as N‑arachidonylethanolamide) (Muniyappa et al., 2013; Pertwee, 2008). CBD has also been found to regulate mesolimbic dopamine activity (Murillo-Rodríguez et al., 2011) and attenuate substance-induced dysregulation of the mesolimbic circuitry (Renard et al., 2016). Consequently, the inclusion of CBD in all forms of legally prescribed medical cannabis provides grounds for a molecular mechanistic defence against dependence. There is some evidence that CBD may play an active role in pain reduction and may lead to a form of psychological dependence. However, this evidence is not consistent across studies (Argueta et al., 2020). Indeed, a recent randomised controlled trial (RCT) of three doses of CBD and matched placebo has found that CBD (400, 800 mg daily over 4 weeks) leads to a reduction in daily cannabis use in men with CUD (Freeman et al., 2020).

#### Medical cannabis: evidence of dependence?

To date, there is very limited research investigating the relationship between medical cannabis use and dependence. There are three aspects of dependence that must be included when holistically considering medical cannabis use, namely: physical, psychological and medical. Physical dependence is characterised by withdrawal symptoms upon cessation of medication use. This occurs due to a physiological adaptation to the drug and the body’s response to compensate for the drug’s actions. The second aspect is psychological dependence which encompasses powerfully motivating cognitive processes that maintain drug consumption to avoid the anticipated negative consequences of stopping use (Nutt, 2003). Dependence as a medical diagnosis attempts to combine both physiological, psychological as well as behavioural aspects to dependence such as drug-seeking behaviours. This perspective allows for a multi-domain analysis of contributing and maintaining factors to dependence. Nonetheless, a nuanced examination of dependence domain-specific effects of medical cannabis is warranted to assess the extent of knowledge in the field and to highlight research gaps.

To date, the clinical assessment of problematic cannabis use has been largely informed by the Cannabis Use Disorder Identification Test short form (CUDIT-SF) (Bonn-Miller et al., 2016) as well as the Cannabis Use Disorder Identification Test revised (CUDIT-R). Both of these were designed to tap problematic use, including dependence, in recreational users. They were not intended nor validated for that purpose. To address this, several of us (HVC, CH, AKS) are currently developing a measure to specifically assess problematic medical cannabis.

Despite this paucity of evidence, the United Nations’ *Report of the International Narcotics Control Board for 2018* declared that dependence is a probable outcome of daily medical cannabis use and that the risk of dependence might be as high as one in three persons (United Nations, 2019). Those using THC related compounds daily (e.g. for chronic pain) may have a greater risk of dependence over those using it weekly for chemotherapy-induced nausea (United Nations, 2019). However, at present there is a lack of data to support these claims and they provide no structure as to how they might be substantiated.

From a scientific perspective to prove evidence of dependence we would need to see:

1. **Increased use over time consistent with tolerance.** This would need to be independent of the intended therapeutic increase of dose.
2. **Significant withdrawal symptoms when the drug is stopped.** There are two withdrawal syndromes that we may see:
(a) Discontinuation syndrome: an appearance of symptoms that were not part of the original disorder for which the CBMP was prescribed include craving, sleep problems, nightmares, anger, irritability, dysphoria and nausea.
(b) Rebound syndrome: an increase in the severity of symptoms of the original condition.
3. **Craving for the drug.** This would be characterised by a loss of control over medication use. Even if medical cannabis may not be pleasurable it may still be craved once use is established, as can happen with other medicines such as opioids.

Table 1 lists the DSM-5 symptoms for recreational cannabis use which consist of previous diagnostic criteria for cannabis dependence and abuse from DSM-IV with the inclusion of the new DSM-5 criteria of ‘craving’. Comparing dependence rates between medical and recreational users, Lin et al. (2016) used these criteria to define past year cannabis abuse or dependence and found that 10% of recreational users and 11% of medical users met the criteria for cannabis dependence. A similar prevalence of cannabis use disorders in people who use cannabis recreationally vs medically was found by Bonn-Miller et al. (2014). Yet looking at the applicability of these symptoms to medical cannabis use, our summary suggests that these criteria may not be the most suitable assessment of potential medical cannabis dependence.

The number of people dependent on cannabis (defined as people using prescribed cannabis outside of their medical dose and treatment regime) may well grow as medicinal cannabis use becomes more acceptable and the drug more accessible. There is conflicting evidence that this is already happening in the USA where some data has shown that adults in states that permit medical use of cannabis have higher rates of daily cannabis use and CUD than states that have not passed this legislation (Hasin et al., 2015, 2017). Conversely, other sources have shown that following medical cannabis enactment laws no significant changes were seen in CUD and there was no significant increase in cannabis consumption in younger age groups. There was however an increase in cannabis use in people aged 26+ years (Mauro et al., 2018) which could be in part due to an increase in medical prescriptions, the majority of which are made for over 30 s (Fairman, 2016).

Interestingly, data from Canada has shown that since the legalisation of recreational cannabis there has been a fall in the number of sales for medical purposes, suggesting some users were accessing medical cannabis for non-medical needs and were now accessing cannabis via the recreational market (Government of Canada, 2019). In spite of this, we argue that medical cannabis diversion in the UK would be an unlikely scenario currently due to the significantly higher cost of medical cannabis compared with illicit supplies and the relatively low ‘rewarding’ effects of licenced CBMPs.

### Differences between medical and recreational users

Medical and recreational cannabis users differ in the motivation and patterns of use of cannabis (Wang et al., 2008; please see Table 2 below). Medical cannabis users often have poorer general health (e.g. depression and anxiety, and registered disability status) than recreational users (Goulet-Stock et al., 2017; Lin et al., 2016). This is unsurprising as individuals using medical cannabis by definition have an underlying medical condition they are addressing through their use.

**Table 2.** Characteristics of medical vs recreational use and users.

Medical use	Recreational use
Often daily (55.9% of users report daily use, 23.5% report weekly use) (Stewart, 2020)	Full range from rarely to daily; only some daily users are addicted (Cuttler and Spradlin, 2017)
Various routes of administration (Borodovsky et al., 2016)	Often smoked with tobacco in the UK (Winstock et al., 2017)
Lower controlled dose of known quantity of cannabinoids/cannabis (Cash et al., 2020)	Higher THC dose, often unknown, cannabinoid content usually unknown (Cash et al., 2020)
Regulated quality (e.g. cGMP)	Unknown quality
Poorer general physical health (Lin et al., 2016)	Usually good general health (Lin et al., 2016)
Poorer psychological well-being (Goulet-Stock et al., 2017)	Generally good psychological well-being (Goulet-Stock et al., 2017)
Lower physical QoL scores	Generally normal range of QoL
Higher age (>50 years) (Turna et al., 2020)	Lower age (Winstock et al., 2019)
Main aim to alleviate symptoms (Stith et al., 2018)	Main aim enjoyment, relaxation, social effects (Geraint et al., 2008)
Little desire to get ‘high’. Represented in a sample of MS patients with high drop out rate from Sativex (THC based medication) (Carotenuto et al., 2020)	Liking effects of THC (Osborne and Vogel, 2008)
Lower prevalence of substance use disorder (including alcohol) (Compton et al., 2017)	Often other psychoactive drug use (Compton et al., 2017)

cGMP: current good manufacturing practice; QoL: quality of life; THC: delta-9-tetrahydrocannabinol; UK: United Kingdom.

It must be acknowledged that the division between medical and recreational cannabis users is blurred at best as many medical users also use non-medically (Choi et al., 2017; Wall et al., 2019) and many recreational users self-medicate conditions such as social anxiety with cannabis.

Chronic pain is the most common condition for which patients seek medical cannabis (Boehnke et al., 2019). Chronic pain is often comorbid with depression and anxiety (Feingold et al., 2019). However, a comprehensive review of placebo-controlled studies of cannabis and CBMPs for pain, found that few assessed abuse liability (Cooper and Abrams, 2019). Feingold et al. (2017) found the prevalence of problematic medical cannabis use to be relatively high, with 21.2% (DSM-IV) and 10.6% (modified Portnoy’s Criteria (PC)) amongst chronic pain patients. Feingold et al. (2019) highlight that severe depression may be one risk factor for medical cannabis dependence among chronic pain patients.

Hasin et al. (2020) found that the prevalence of non-medical cannabis use and CUD was higher amongst people with chronic pain across a 10-year period in the USA. Problematic use was more common in patients using larger amounts for longer periods, reporting higher levels of depression or anxiety and using other drugs including alcohol. However, comparing the same population’s problematic opioid use, problematic use of prescription opioids was far more prevalent than problematic medical cannabis use (Feingold et al., 2017).

It is important to interpret the (limited) evidence with caution and within its wider context. Dependence rates for medical users have to be contextualised firstly in terms of other drug use, (e.g. less dependence on more harmful drugs such as opioids (Feingold, 2017)) and secondly in terms of the patient’s medical condition (e.g. if a chronic condition is successfully treated by medical cannabis, a degree of dependence might well be acceptable to users). It is important not to confuse the desire to continue taking a drug that treats chronic symptoms which re-emerge when the drug is stopped, with the craving of addiction: paracetamol for instance can be used repeatedly to treat chronic pain (Singh et al., 2017).

Although medical cannabis use may lead to dependence, it compares favourably in regards to safety and tolerability with other drugs, such as opioids, and it is important to weigh up the benefits of use vs the risk of dependence. Many of the conditions for which medical cannabis is used are chronic, highlighting the need for a medicine with a good safety profile. Especially for chronic pain patients, adverse effects may only play a limited role, even tolerance or dependence. Other medications currently used for chronic pain, such as pregabalin and opioids, have a higher potential for abuse and dependency, and may cause more severe withdrawal symptoms after cessation than medical cannabis (Edlund et al., 2014). When prescribing, physicians need to take this trade-off into consideration, particularly when use is long-term. In their long-term observational study of 180 chronic pain patients, Takakuwa et al. (2020) found that medical cannabis can indeed diminish prescription opioid usage, working as a preferred alternative to prescription opioids in over half of their patients.

The issue of CUD has become more relevant in relation to other emerging trends in drug dependence particularly the current opioid crisis in the USA. Here, there is a nationwide federal focus on prescription opioid harm reduction strategies, that includes alternative therapies for pain management, such as medical cannabis. There is a developing evidence base that some chronic pain patients are able to substitute opioids with medical cannabis (e.g. Bergeria et al., 2020; Takakuwa et al., 2020; Vyas et al., 2018). Furthermore, Piper et al. (2017) identified a reduction in opioid overdose deaths in those US states that legalised medical cannabis. In this arena it is vital that appropriate definitions of addiction and dependence to opioid substitute medicines are followed to avoid conflating opioid dependence with medical cannabis use.

Similarly, medical cannabis users often also have lower levels of alcohol-use disorders and non-cannabis drug use severity compared to recreational users (Lin et al., 2016; Wall et al., 2019; Woodruff et al., 2016) which might indicate that medical cannabis use may reduce (‘substitute’ for) other psychoactive substance use, including alcohol (Bonn-Miller et al., 2014; Loflin et al., 2017; Reiman, 2007). Again, relative dependence potential of the different drugs needs to be taken into account.

### Dose

One of the primary issues with the potential for medical cannabis dependence is dosage. Since a standard dose or unit of cannabis does not yet exist, patients are often required to titrate their dose to their desired effect while trading off any negative effects. The patient rather than the doctor determines the correct dose. Unlike with standardised preparation of CBMPs, smoked flower is harder to titrate and quantify, especially for non-experienced users as demonstrated by studies in recreational users (Freeman et al., 2014). Several conceptual units have been developed for recreational cannabis use (Hindocha et al., 2018; Kögel et al., 2017). For example, Freeman and Lorenzetti (2019) define a standard unit as 5 mg of THC whilst Chester et al. (2020) suggest that a standard unit should be the THC amount corresponding to the lowest dose that produces subjective effects.

When patients are prescribed Sativex, each 100 µL spray contains 2.7 mg THC and 2.5 mg CBD, 0.04 mg ethanol and very small amounts of other cannabinoids (GWPharma, 2019). Thus, in its formulation and composition Sativex is very different to cannabis. The prescribing guidelines suggest that patients gradually titrate their dose (i.e. sprays per day) for the first two weeks, from 1–12 if needed. In routine clinical practice, the median number of sprays is four (equivalent to 10.8 mg THC and 10 mg CBD), and in clinical trials, it was nine sprays. Therefore, just as one might self-titrate how much cannabis one inhales, the guidelines are defined to ensure patients can self-titrate to the amount they need.

A GW-Pharma sponsored study designed to identify the abuse liability of Sativex in recreational cannabis users compared four sprays, eight sprays and 16 sprays (10.8, 21.6 and 43.2 mg THC, respectively) of Sativex with two doses of dronabinol (20 mg and 40 mg) and placebo in a randomised order (Schoedel et al., 2011). Four sprays (10.8 mg) of Sativex showed no more abuse liability (‘drug liking’, euphoria, subjective drug value) than placebo. Higher doses of eight and 16 sprays showed a greater liability for abuse than placebo. However, equivalent doses of dronabinol showed greater abuse liability than Sativex (Schoedel et al., 2011). Therefore, higher doses of dronabinol and Sativex are associated with abuse-related subjective effects. Moreover, in one study of cannabinoid replacement therapy, both dronabinol and Sativex had higher self-reports of liking than placebo (Allsop et al., 2015).

It should be noted that in these studies, the entire dose was given at once, whereas in routine clinical practice, single sprays are used throughout the day. Moreover, Schoedel et al.’s (2011) study was conducted with recreational cannabis users who could use cannabis in between sessions and are more likely to respond to different subjective effects than non-cannabis experienced patients with clinical disorders. For neither drug has abuse been observed in post-marketing research (Calhoun et al., 1998; Robson, 2011; Wade et al., 2006).

### Frequency of use

A key issue in the debate of problematic (recreational) cannabis use, is the role of frequency of use because many people who use cannabis daily experience no clinical problems (Braidwood et al., 2018). Regarding medical use, patients often are titrating up to a higher dosage than recreational cannabis users, as they may be using throughout the day (Goulet-Stock et al., 2017; Roy-Byrne et al., 2015). Although daily cannabis use can be associated with a range of other risk factors (Hughes et al., 2014) it is not inherently problematic, especially when treating a chronic condition.

The indicators of frequency and dose, often used to indicate recreational cannabis dependence, will not necessarily capture medical cannabis dependence as it is important to ’resist the temptation to assume that daily consumption is implicitly problematic’ (Asbridge et al., 2014, p. 263). As an example of this, when validating the CUDIT-SF (Bonn-Miller et al., 2016), 100% of medical cannabis patients endorsed using cannabis every day. Cannabis dependence in this population was best predicted by four CUDIT items: not being able to stop using cannabis once you had started, spending a great deal of time getting, using and recovering from cannabis, and memory/concentration impairment. The internal consistency of the CUDIT-SF was 0.66 for medical patients in the USA and 0.8 in recreational users. In another analysis validating the CUDIT-R (the revised version, from which the CUDIT-SF was derived) in veterans using medical cannabis, Loflin et al. (2018) found that the single factor model of the CUDIT-R previously validated in recreational users only accounted for 38% of the variance for medical users. These studies suggest that current measures of dependence are not necessarily appropriate for medical cannabis users.

### Potency and THC: CBD ratio

Prolonged use of high-potency cannabis has been associated with dependence and mental health problems in recreational users (Arterberry et al., 2019; Di Forti et al., 2016, 2019; Freeman and Winstock, 2015; Freeman et al., 2018). ’High potency’ is commonly judged in research as >10% THC. In the medical cannabis literature, higher potency has been associated with more adverse events (Andreae et al., 2015; Wallace et al., 2015; Wilsey et al., 2013). Higher CBD levels in cannabis have been shown to ameliorate cognitive, psychotic- and dependence-related effects of THC (Curran et al., 2016; Englund et al., 2013; Morgan et al., 2010, 2012). A recent comparison of THC and CBD levels available in recreational and medical markets in the USA show that the average THC concentration available in medicinal programmes was 19.3%, similar to recreational programmes where it was 21.5% (Cash et al., 2020). CBD levels, in contrast, were 2% and 1.3% in medical and recreational programmes, respectively (Cash et al., 2020). Given that medical cannabis users are more likely to use cannabis daily, a case could be made for limiting THC in medical cannabis as seen in the Uruguayan market where cannabis sold in pharmacies is under 10% THC (EMCDDA, 2020).

Ratios of THC: CBD can impact the therapeutic potential, metabolism and side effects of the drug. Direct comparisons of CBMPs suggests that an acute combination of THC: CBD (Sativex) produce less abuse liability effects than synthetic THC (dronabinol) at the same dose (Schoedel et al., 2011). A recent systematic review (Freeman et al., 2019b) suggests some studies have found CBD can reduce intense experiences of anxiety or psychosis-like effects of THC and blunt potential impairments on emotion and reward processing. However, these effects were not consistently observed and there were high levels of heterogeneity in dose, route and ratio. Importantly this review was of studies with recreational and not medicinal cannabis users. Another recent systematic review reported 11 studies investigating a range of THC:CBD ratios but no clear conclusions could be drawn about dose effects or about the best ratio for safest therapeutic use (Zeyl et al., 2020). Overall, the limited literature suggests that the role of THC:CBD ratio in medical cannabis dependence is not yet clear.

### Pharmacokinetics and route of administration

Pharmacokinetics, the process by which drugs move through the body, is dependent on the route of administration of cannabis. These processes are dynamic, potentially changing over time, and likely affected by the frequency and magnitude of drug exposure. Smoking cannabis is the most common route of administration for recreational users (Hindocha et al., 2016) but is not recommended for medical use because of respiratory concerns. Thus, vaporisers have been used as an alternative. In a placebo-controlled crossover trial in healthy adults who infrequently used cannabis, Spindle et al. (2018) report a comparison of acute effects of smoked vs vaporised cannabis at two doses. They found higher THC concentrations in whole blood following vaporised vs smoked cannabis at both the 10 mg and 25 mg doses and these demonstrated a dose-related. Such research suggests vaporising may lead to stronger effects than smoking.

Bioavailability of the oral route of cannabinoids is about 6% (much lower than smoked/vaporised administration), and this is likely because of degradation of the drug in the stomach and significant levels of first-pass metabolism in the liver. The oral route may also reduce the risk of problematic cannabis use although this has not directly been investigated. A comparison of pharmacokinetic parameters can be found in Tables 3 and 4.

**Table 3.** Pharmacokinetics (mean C_max_ and T_max_) of tetrahydrocannabinol (THC) and cannabidiol (CBD) in occasional (non-current) users, in blood, after three doses of THC and one dose of CBD.

	Smoked	Vaporised	Oral
THC (10 mg) (Spindle et al., 2018 – smoked and vapourised; Vandrey et al., 2017 – oral)			
C_max_ (ng/mL)	3.76	7.53	1^a^
T_max_ (h)	0.11	0.18	0.9^a^
THC (25 mg) (Spindle et al., 2018 – smoked and vapourised; Vandrey et al., 2017 – oral)			
C_max_ (ng/mL)	10.24	14.36	3.5^a^
T_max_ (h)	0.13	0.19	2.6^a^
THC (50.6 mg) (Newmeyer et al., 2016)			
THC C_max_ (ng/mL)	51.6	47.8	10.3^a^
THC T_max_ (h)	0.11	0.11	2.3^a^
CBD (100 mg) (Spindle et al., 2018)			
CBD C_max_ (ng/mL)	181.4^b^	104.6	11.1
CBD T_max_ (h)	0.1	0.1	3

a Oral via a brownie.

b Smoked a high-CBD-dominant strain, which had 100 mg CBD and 3.7 mg THC.

**Table 4.** Pharmacokinetics (mean Cmax and Tmax) of a single dose of Sativex, Dronabinol and Epidiolex.

	THC C_max_	THC T_max_	CBD C_max_	CBD T_max_
Sativex (5.4 mg THC and 5 mg CBD oromucosal spray; Karschner et al., 2011)	5.1	3.3	1.6	3.7
Dronabinol (5 mg capsule; Parikh et al., 2016)	2.2^a^	1^a^		
Epidiolex (1500 mg oral solution; Taylor et al., 2018)			292.4	4.0

a Article reports dronabinol pharmacokinetics parameters.

Sativex is an oromucosal spray which has a slower onset than smoking or vaporising cannabis (GWPharma, 2005, 2019). When this method was compared to oral THC (dronabinol) at the same dose, the pharmacokinetic profiles differed (see Tables 3 and 4). Dronabinol had a greater peak plasma THC concentrations with 1.5 h post-administration as well as a more rapid decline in comparison with Sativex (Schoedel et al., 2011). Sativex is likely to have more than one peak (T_max_) because of early buccal administration followed later by gastrointestinal absorption. There is also greater inter-subject variability between participants. Moreover, both drugs produced lower peak concentrations than smoked cannabis (3.55% THC) (Huestis et al., 1992) and lower subjective effects related to abuse liability (e.g. like drug effect, euphoria) (Cooper et al., 2013).

Together these studies suggest that oral administration routes such as buccal or swallowing is preferred as it could reduce the risk of abuse-related subjective (and neurocognitive) effects (Cooper and Abrams, 2019). However, potential misuse and problematic use of oral cannabinoids have been found in a prospective observational study of 265 patients initiating oral cannabinoid therapy. Ware et al. (2018) compared three measures (Current Opioid Misuse Measure (COMM); Addiction Behaviour Checklist, Chabal Prescription Opioid Abuse Checklist) modified by replacing the word ’opioid’ with ’cannabinoid’. At follow-up, up to a quarter of patients reported problematic prescription oral cannabinoid use on the COMM (Ware et al., 2018). This is important because the COMM is a self-reported and relatively detailed measure of dependence whilst the other measures are short, clinician-rated measures. This potentially suggests that patients have greater worries about medical cannabis dependence than do their clinicians.

Intrapulmonary administration of cannabinoids (e.g. by smoking or vaporising) is considered to be an effective route of administration given its high systemic bioavailability, fast onset of action, short duration of peak effects, and limited duration of effects relative to other methods (e.g. oral or buccal). Moreover, efficient, precise and consistent vaporization delivery systems are under development (Almog et al., 2020). Short drug durations may play a role in medical cannabis dependence in that frequency of use would be greater. Furthermore, rapid drug delivery via smoked or vaporised forms of THC have been linked to greater reinforcement and addiction-related effects for example by promoting forms of neurobehavioural plasticity that contribute to compulsive drug-seeking (Samaha and Robinson, 2005).

### Set and setting: the importance of context

Set, defined as mindset factors (e.g. personality, preparation, expectation and intention of the person involved) and setting, defined as the physical, social and cultural environment in which the drug use takes place (Hartogsohn, 2017), are likely to have an impact of the addictive potential of CBMPs because the result of cannabis consumption is not a linear function of individual dose and dosing (Asbridge et al., 2014). It is possible to consume cannabis regularly and heavily and not experience CUD and current theories of addiction do not address this observation (Temple et al., 2011).

Set and setting are often defined as the non-drug parameters of psychopharmacology (Feldman, 1963) and are often not investigated because including them in clinical drug development would lead to highly complicated trials. Although set and setting are most often discussed in relation to psychedelic research and recreational drug use, its importance for CBMPs should also be investigated. For example, in three studies investigating problematic medical cannabis use, those with a history of psychiatric problems or other drug-use problems (including alcohol), tobacco smokers and recreational cannabis users were more likely to experience medical cannabis dependence (Feingold et al., 2017; Ware et al., 2018). Moreover, in regards to recreational cannabis dependence, van der Pol et al. (2013) found strong evidence pointing to increased risk for CUD among individuals who use cannabis in order to cope with mental distress. Furthermore, the mindset factors for recreational cannabis include the desire to experience the euphoric effects of the drug, whilst for medical cannabis users this is not the desire, indeed many patients discontinue medical use due to these subjective effects (Carotenuto et al., 2020).

Setting differs between medical and recreational cannabis use as well. Medical cannabinoids may be administered in hospitals or other medical environments, whilst recreationally people use cannabis in many different environments. The line between recreational and medical becomes blurred in the home, a common place for both medical and recreational administration. Socio-cultural factors may also be at play, for example, in those using black-market cannabis for medical use, the drug may have more harmful implications due to stigma-related factors in comparison to legal, regulated markets.

The context of medicinal and recreational use differ significantly which will have a major impact on the development and maintenance of dependence. The seminal work of Lee Robins and her colleagues (Robins, 1973; Robins et al., 1974) on heroin addiction among Vietnam veterans shows that many of the key factors in the development and maintenance of addiction go well beyond the simple pharmacology of the drug. These factors were price, availability, how the drug is delivered, the availability of other substances, social norms, education and life circumstances (Hall and Weier, 2016). Robins’ studies found high rates of heroin use (34%) and symptoms of heroin dependence (20%) among US soldiers while serving in Vietnam. In the first year after returning to the USA, only 1% became re-addicted to heroin, although 10% had tried the drug again.

## Discussion

There are notable differences between medical and recreational cannabis users in terms of routes of administration, doses, forms of cannabis as well as set and setting factors. Each of these can significantly affect the potential for dependence. However, there are other factors that we have not explored in this review that require further investigation such as age, ethnicity, cultural factors and concurrent medications. This makes it unjustifiable to directly extrapolate findings from recreational use to medical use. Also because of these differences, the tools traditionally used to assess recreational cannabis dependence are less relevant to measure medical cannabis dependence. Basic research on the prevalence and correlates of medical cannabis dependence is lacking in this new research field which comprises a patchwork of policies regulating medical and recreational use across the globe.

There are still many uncertainties related to medical cannabis and its dependence potential due to the lack of scientific research which resulted from the previous Schedule I status of the drug. After being neglected in the second half of last century, today medical cannabis is increasingly becoming part of the normal pharmacopeia. The development of future research investigating the broad variety of benefits as well as risks associated with the medical use of cannabinoids is essential. It is important to prepare in advance so that issues related to dependence can be addressed in a timely and efficient manner and minimised through effective risk assessment, risk management and policy making. The risks of neglecting this research are evidenced by the tragic- and largely preventable-opioid epidemic in the USA. In relation to medical cannabis, there are a range of key recommendations which can be implemented to help increase/ensure patient safety (see Panel 1).

**Panel 1.** Key recommendations.

1. THC: CBD ratio
As THC in higher doses is associated with dependence whilst CBD appears to have anti-dependence properties it is vital to optimally balance the cannabinoid content of CBMPs to potentially block the development of dependence.
2. Ensure safe supply
It is important that patients are able to access a reliable medical cannabis product rather than having to rely on the black market with its risks and inconsistent products.
3. Develop safer use guidelines
Lower daily cannabis use is associated with better clinical profiles as well as safer use behaviours, i.e. preference for CBD and non-inhalation administration routes. This highlights the importance of developing cannabis use guidelines for clinicians to better protect their medical cannabis patients. Evidence-based Lower-Risk Cannabis Use Guidelines (LRCUG) already exist in relation to recreational use (Fischer et al., 2017) and it is imperative to develop these factors focusing specifically on the medical use of cannabis. Risk of dependence might then be mitigated with these factors in mind by physicians giving harm reduction advice and clear clinical guidelines.
4. Screening of patients
It is important to screen for factors which may make an individual more vulnerable to becoming dependent of CBMPs (e.g. previous Substance Use Disorder (SUD)s; current heavy recreational cannabis use). Treatment providers may also need to assess for other (mental) health conditions (e.g. depression and anxiety) when prescribing medical cannabis.
5. Monitoring of use
Potential harms have to be managed e.g. through monitoring (Schlag et al., 2020). Regular monitoring of patients’ medical cannabis use is important to stem the development of dependence. This may be done through using a patient registry such as Project TWENTY21 (T21).
6. Personalised medicine
Medical cannabis is a part of the development towards more personalised medicine, and THC: CBD ratios, frequency of use, routes of administration, and subsequent dependence risks will likely differ between conditions and patients. It seems likely that genetic variations in cannabis receptors and metabolism will also be relevant.
7. Balancing patient need and potential for harm
It is essential to balance patient need and the potential for harm. Any risk of medical cannabis dependence needs to be weighed up in relation to alternative medications, some of which have potentially higher abuse liabilities. When prescribing, physicians and patients together need to take this trade-off into consideration, particularly when use is likely to be long-term. It is important to educate patients on the potential risk for dependence and withdrawal. Strategies for reducing dependence (e.g. ‘drug holidays’ like with methylphenidate treatment; increasing CBD and reducing THC dosages) would need evaluating.

### Limitations and future research

Not enough is known about medical cannabis users, many of whom are chronic users. Longitudinal data are needed to investigate trajectories of use and potential dependence. Medical users who transition from the black market in comparison to those who begin only when a regulated market was initiated are likely significantly different groups that should be included in the clinical research. Future studies need to examine patient outcomes longitudinally to further understand potential risks including risk factors for dependence.

As most of the research on cannabis dependence has been conducted in relation to recreational use, to date no specific scale exists to assess medical cannabis dependence. With the increasing use of medical cannabis globally, the development of such a scale is vital. Co-authors of this article (HVC/CH) are currently developing the Cannabis-Based Medicines Questionnaire (CBM-Q) to specifically address problematic medical cannabis use. This questionnaire will be utilised in Project TWENTY21 (the largest UK registry of medical cannabis patients: https://drugscience.org.uk/project-twenty21/), with a high number of respondents over a longitudinal timeframe and the assessment of potential links to predisposing factors, such as the underlying medical condition/s. Tracking dependence is challenging and needs to occur in the context of clinical trials as well as careful monitoring of real-world experiences.

## Supplemental Material

sj-pdf-1-jop-10.1177_0269881120986393 – Supplemental material for Cannabis based medicines and cannabis dependence: A critical review of issues and evidence Click here for additional data file. Supplemental material, sj-pdf-1-jop-10.1177_0269881120986393 for Cannabis based medicines and cannabis dependence: A critical review of issues and evidence by Anne K Schlag, Chandni Hindocha, Rayyan Zafar, David J Nutt and H Valerie Curran in Journal of Psychopharmacology
