# Supplementary material for: Cannabis based medicines and cannabis dependence: A critical review of issues and evidence
Source: J Psychopharmacol. 2021 Feb 17;35(7):773–85. doi: 10.1177/0269881120986393 (PMC8278552; doi:10.1177/0269881120986393)
Supplement: sj-pdf-1-jop-10.1177_0269881120986393 – Supplemental material for Cannabis based medicines and cannabis dependence: A critical review of issues and evidence [file sj-pdf-1-jop-10.1177_0269881120986393.pdf]

SUPPLEMENTARY MATERIALS

Cannabis based medicines and cannabis dependence: a critical review of issues and evidence

Anne K Schlag[1,5], Chandni Hindocha[2,3,4], Rayyan Zafar[1,5], David Nutt[1,5] & H Valerie Curran[2,4]

[1] Drug Science
[2] Clinical Psychopharmacology Unit, University College London, WC1E 7HB
[3] Translational Psychiatry Research Group, Research Department of Mental Health Neuroscience, Division of Psychiatry, Faculty of Brain Sciences, University College London
[4] University College Hospital National Institute of Health Research (NIHR) Biomedical Research Centre
[5] Neuropsychopharmacology Unit, Centre for Psychiatry, Division of Brain Sciences, Imperial College London, London, UK

Title	Year	Authors	Main results
Abuse potential of dronabinol (Marinol®)	1998	Calhoun S, Galloway G, Smith D.	No evidence of diversion. Overwhelming majority of user reports indicate that its effects are dysphoric and unappealing. This profile of effects gives dronabinol a very low abuse potential.
The adverse effects of cannabinoids: implications for use of medical marijuana *	2008	Louisa Degenhardt, Wayne D. Hall	Cannabis dependence can develop, but the evidence on risk factors for dependence comes primarily from studies involving recreational users who began using cannabis in adolescence and early adulthood and who use the most potent products. These users smoke cannabis with a greater frequency and intensity than older adults who would use smaller doses for symptom relief. “We know nothing of the risks of incident cannabis dependence in the context of long-term, supervised medical use.”
Abuse potential and psychoactive effects of δ-9-tetrahydrocannabinol and cannabidiol oromucosal spray (Sativex), a new cannabinoid medicine	2011	Robson P.	Tolerance did not occur. Abrupt withdrawal did not result in a formal withdrawal syndrome. No cases of abuse or diversion were reported. Abuse or dependence on Sativex likely to occur only in a very small proportion of recipients.
Cannabinoids for nausea and vomiting in adults with cancer receiving chemotherapy	2015	Lesley A Smith, Fredric Azariah, Verna Lavender, Nicola Stoner, Silvan Bettiol	Participants had more chance of reporting 'feeling high' (3 trials; 137 participants; RR 31; 95% CI 6.4 to 152). The percentage of the variability in effect estimates that is due to heterogeneity rather than chance was not important.
A randomized, double-blind, placebo-controlled, crossover study to evaluate the subjective abuse potential and cognitive effects of nabiximols oromucosal spray in subjects with a history of recreational cannabis use	2016	Schoedel K, Chen N, Hilard A, White L, Stott T, Russo E et al.	Both dronabinol and nabiximols had significant abuse potential compared with placebo at higher doses. The abuse potential of nabiximols was no higher than that of dronabinol.
Comparing adults who use cannabis medically with those who use recreationally: Results from a national sample	2016	Lin L, Ilgen M, Jannausch M, Bohnert K.	A third of medical cannabis users had daily cannabis use compared to 11% of recreational users. 11% of medical users and 10% of recreational users met criteria for abuse/dependence.

Problematic use of prescription opioids and medicinal cannabis among patients suffering from chronic pain	2017	Feingold D, Goor-Aryeh I, Bril S, Delayahu Y, Lev-Ran S.	Problematic use of opioids and cannabis was more common in patients using medications for longer periods of time, reporting higher levels of depression and anxiety, and using alcohol or drugs. Problematic use of cannabis was more common amongst individuals using larger amounts. Problematic use of prescription opioids is more prevalent than problematic use of medical cannabis.
Dronabinol is a safe long-term treatment option for neuropathic pain patients	2017	Schimrigk S, Marziniak M, Neubauer C, Kugler E, Werner G, Abramov-Sommariva D.	No signs of drug abuse and only one possible case of dependency occurred.
A prospective observational study of problematic oral cannabinoid use	2018	Ware M, Martel M, Jovey O, Lynch M, Singer J.	Approximately 25% of patients prescribed oral cannabinoid therapy demonstrate problematic medical cannabis use. Risk of problematic use was higher for patients with psychiatric problems.
Mental health functioning and severity of cannabis withdrawal among medical cannabis users with chronic pain	2019	Perron B, Holy K, Yeagly E, Ilgen M.	67.8% reported at least one moderate or severe withdrawal symptom. Patients with low mental functioning had higher rates of withdrawal symptoms than patients with high mental functioning Cannabis withdrawal symptomology is highly prevalent in patients using at least 3x/week.
Medical marijuana dependence among chronic pain patients suffering from depression and anxiety	2019	Feingold D, Bril S, Goor-Aryeh I, Delayahu Y, Lev-Ran S	Participants with severe depression were significantly more likely to screen positive for cannabis dependence than those without depression.

*Key words used for Pub Med Search:
'Medical' and 'Cannabis or Marijuana' and 'Dependence or Abuse or Addiction' Searched April 21st 2020

*Key words used for Cochrane Library Search:
'Medical' and 'Cannabis or Marijuana' and 'Dependence or Abuse or Addiction' Searched May 13th 2020

Supplementary Table 1: Studies of the dependence and abuse potential of cannabis-based medicines
